# Compensation of Trial-to-Trial Latency Jitter Reveals the Parietal Retrieval Success Effect to be Both Variable and Thresholded in Older Adults

**DOI:** 10.3389/fnagi.2019.00179

**Published:** 2019-07-23

**Authors:** Jamie G. Murray, Guang Ouyang, David I. Donaldson

**Affiliations:** ^1^Department of Psychology, Faculty of Natural Sciences, University of Stirling, Stirling, United Kingdom; ^2^The Laboratory of Neuroscience for Education, Faculty of Education, The University of Hong Kong, Hong Kong, China

**Keywords:** cognitive aging, episodic recollection, parietal ERP effect, retrieval accuracy, residual iteration decomposition analysis

## Abstract

Although the neural mechanism supporting episodic recollection has been well characterized in younger adults, exactly how recollection is supported in older adults remains unclear. The electrophysiological correlate of recollection—the parietal retrieval success effect—for example, has been shown to be sensitive to both the amount of information recollected and the accuracy of remembered information in younger adults. To date, there is mixed evidence that parietal effect also scales with the amount of information remembered in older adults whilst there is little evidence that the same mechanism is sensitive to the accuracy of recollected information. Here, we address one potential concern when investigating Event Related Potentials (ERPs) among older adults—namely, the greater potential for single-trial latency variability to smear and reduces the amplitudes of averaged ERPs. We apply a well-established algorithm for correcting single-trial latency variability, Residual Iteration Decomposition Analysis (RIDE), to investigate whether the parietal retrieval success effect among older adults is sensitive to retrieval accuracy. Our results reveal that similar to younger adults, older adult parietal retrieval success effects scale with the accuracy of recollected information—i.e., is greater in magnitude when recollected information is of high accuracy, reduced in magnitude when accuracy is low, and entirely absent when guessing. The results help clarify the functional significance of the neural mechanism supporting recollection in older adults whilst also highlighting the potential issues with interpreting average ERPs in older adult populations.

## Introduction

Retrieval of specific events from one’s recent and distant past is a defining feature of human episodic memory, reflected in the experience of “recollecting.” Episodic recollection can be distinguished from other memory retrieval processes, such as the recovery of semantic information (i.e., “Chicago is commonly referred to as the windy city”) or familiarity (i.e., recognizing someone but not being able to remember anything more about them: for a review, see Yonelinas, 2002). Recollection is particularly sensitive to age-related cognitive decline with behavioral evidence showing that older adults, relative to younger adults, exhibit a reduction in the amount of information remembered (for a review, see Old and Naveh-Benjamin, 2008) and recovery of less accurate information when remembering is successful (Nilakantan et al., 2018). More qualitative measures of episodic memory (such as the autobiographical interview—Levine et al., 2002) also suggests that healthy aging is associated with a shift in the focus of retrieval towards the recovery of more general and gist based information. Whilst behavioral evidence suggests an age-related functional change in recollection, there is uncertainty about how the neural mechanisms supporting episodic recollection operate in older adults. Below, we outline a continuous source task that allows us to directly assess the accuracy of episodic recollection responses whilst recording brain activity, revealing that the electrophysiological correlate of recollection operates in much the same way as in younger adults—i.e., as reflecting a some-or-none thresholded process.

**Figure 1:**
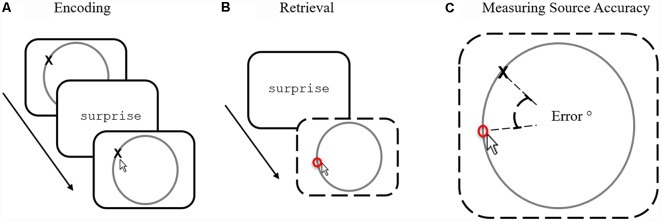
The source memory task. **(A)** During encoding participants are instructed to memorize words paired with locations, indicating the location after each trial to confirm attention. **(B)** During retrieval, participants are shown previously presented words and are asked to recall the position using the mouse. **(C)** Source accuracy is measured by calculating (in degrees) between true and responded locations.

Although successful recollection is traditionally defined in quantitative terms (i.e., how much information one can remember about a prior episode), successful recollection also provides rich qualitative information that can be very accurate in some instances and very inaccurate in others. Measuring the quality of memory accuracy has, however, proved difficult because traditional measures rely on subjective ratings (such as confidence ratings or self-reports)—that can be noisy and imprecise. An alternative approach to measuring retrieval quality is to use memory paradigms that objectively and directly measure the accuracy of remembered source information reported during retrieval (Harlow and Donaldson, 2013; Murray et al., 2015; Harlow and Yonelinas, 2016; Richter et al., 2016; Cooper et al., 2017). Here, single words (or pictures) are associated with specific locations around a circle (see Figure 1A) that provide to-be-remembered source information. At test, words (or pictures) are shown again and participants must recover the precise location (Figure 1B) previously associated with the item. Accuracy (or “precision” as it is often referred to) is measured by calculating the degree of error between the reported response location and the actual target location—allowing a fine-grained assessment positional response accuracy (Figure 1C) to be calculated. Studies employing such continuous source paradigms have consistently revealed a pattern of error responses that can be best described as a mixture of guessed trials (whereby recollection fails to return any information about the location) and recollected trials (that vary in location accuracy—i.e., different degrees of error).

Critically, the pattern of error responses reveals important information about the nature of recollection. Traditionally, for example, recollection has been modeled either as a thresholded processes (whereby recollection either succeeds or fails to retrieve information from memory; Yonelinas, 1997) or as a continuous process whereby memory strength varies in a Gaussian fashion from very weak to very strong responses (Wixted, 2007; Slotnick, 2013). Both accounts make clear predictions about the response patterns observed using the continuous source task. If recollection was thresholded, for instance, the pattern of response errors would be a mixture of trials where recollection failed to bring back information from memory (resulting in a randomly distributed response pattern relative to the target) and trials where recollection was successful (i.e., trials that cluster close to the target). In contrast, if episodic memory is defined as a continuous process, we would expect to see only a single distribution of responses, with a higher degree of moderate trials. To date, model fitting of the response errors carried out over a series of studies (see Harlow and Donaldson, 2013; Murray et al., 2015; Harlow and Yonelinas, 2016; Richter et al., 2016) consistently shows that the thresholded and variable account of recollection provides a significantly better fit to the data than the alternative continuous model. Although the strength of evidence in support of the thresholded model of recollection is compelling among younger adults, it is still relatively unclear whether recollection may operate in a similar fashion in older age.

In a recent study by Nilakantan et al. (2018), a similar paradigm was used to measure retrieval accuracy among older adults whereby images were associated with discrete locations in a four by four grid presented on a screen. Performance on the task revealed that older, compared to younger adults, remembered information with less accuracy (i.e., greater distance between the study and target location). In contrast, the study did not reveal any significant change in the amount of information remembered between age groups; a surprising result given the ubiquity of retrieval deficits reported in studies examining age-related differences in recollection (for a review, see Koen and Yonelinas, 2014). It is, however, unclear whether the restricted number of locations used in this study provides sufficient resolution to allow differences in location accuracy to be observed, or whether alternative models of recollection (such as a continuous Gaussian model) could have provided a significantly better fit to the response patterns observed. Moreover, whilst the data from Nilakantan et al. (2018) successfully demonstrates that retrieval of location accuracy is affected by healthy ageing, the results do not address whether recollection operates in a similar way between younger and older adults—i.e., whether recollection remains a variable and thresholded process.

To directly address the question of whether recollection remains a thresholded processes in older age, here we assess the neural correlate of recollection using the continuous source task shown in Figure 1. The use of both neural and behavioral data is critical because if similar response patterns are observed between age groups, from a theoretical perspective, it is nonetheless reasonable to propose that the same behavioral patterns stem from different neural processes (particularly in light of potential compensatory mechanisms that may support memory among older adults—for a review, see Friedman, 2013). Here, we employ the most widely studied neural correlate of recollection—the so-called “Parietal old/new effect,” measured using Event Related Potentials (ERPs) recorded during performance on episodic recognition tasks. Typically, when words are successfully recollected during a recognition memory test, the associated ERP waveform exhibits a positive going shift (relative to ERPs for misses or correctly rejected new items). The resulting “old/new” difference typically onsets around 500 ms post-stimulus and lasts for around 300–400 ms, generally exhibiting a clear Left Parietal distribution (although the topography is less consistent for other populations—see below for further discussion).

Importantly, the Left Parietal effect has been found to vary with the accuracy of recollection, measured using the continuous source paradigm described above. Murray et al. (2015), for example, compared waveforms associated with different degrees of accuracy to a Baseline condition (i.e., responses that were over 90° from the target and therefore reflected the complete absence of remembered information). Murray et al. (2015) observed that trials with high accuracy (defined as trials with a response error of less than 10°) were significantly more positive going than low accuracy trials (ranging from 11° to 35°), whilst trials associated with very weak or guessed responses (i.e., from 35° to 90°) did not significantly differ in amplitude to the Baseline^1^. In short, the pattern of neural data supports the conclusions from behavioral studies in demonstrating that the size of recollection-related ERP effect was sensitive to retrieval accuracy when recollection was successful but was absent when recollection failed to return information from memory (i.e., a thresholded process).

Although the neural mechanism supporting recollection in younger adults showed a clear thresholded pattern, it cannot be assumed that the same pattern will be observed in older adults. One particular problem is that evidence linking the Left parietal old/new effect with recollection is far from consistent within the ageing literature. For example, studies employing words and pictures during standard old/new recognition procedures provide some evidence of parietal retrieval success effects in older adults (Gutchess et al., 2007; Wolk et al., 2009; Wang et al., 2012)—whereas other studies employing similar procedures fail to observe any significant recollection effect at all (Nessler et al., 2007; Ally et al., 2008; Guillaume et al., 2009). Many of these studies cannot be interpreted definitively given that they fail to employ any behavioral measure of familiarity or recollection, making it difficult to associate changes in amplitude or topography to any specific retrieval processes (although exceptions do exist, such as the separation of responses according to judgments of confidence by Wang et al., 2012). Instead, more definitive results can be obtained from ERP studies that are designed to require greater contributions of recollection than familiarity, such as source retrieval or associative recognition paradigms.

Data from source memory experiments, in particular, require participants to successfully recollect contextual information associated with items at encoding (such as the position of a target or its internal/external features). ERP studies of younger adults typically show that the Left Parietal effect is larger in amplitude for source correct than source incorrect judgments—consistent with the view that correct source memory requires recollection (Wilding, 1999). Within ageing studies, similar patterns of neural activity are observed across parietal regions among older adults, albeit with a slightly more right, as opposed to left, asymmetry (see Wegesin et al., 2002; Li et al., 2004; Duverne et al., 2009). Li et al. (2004) have argued that the similar pattern of parietal effects across the right hemisphere (i.e., greater amplitude for source correct than source incorrect judgments) more than likely reflects similar retrieval processes to those observed in younger adults (for further discussion, see Friedman, 2013).

Interpretation of memory-related parietal effects in older adults, however, is complicated by the failure among other source memory studies to observe any significant parietal activity in older adults (see both Swick et al., 2006; Eppinger et al., 2010). Similarly, among associative recognition studies (in which recollection is required to discriminate between intact and re-arranged stimulus pairs) have also failed to observe any significant recollection based effects either specifically across parietal electrodes (Bridger et al., 2017) or across the entire scalp (Kamp and Zimmer, 2015). Instead, a large left-frontal negativity is often observed and has been interpreted as reflecting “compensatory” mechanisms that may operate to counteract age-related recollection deficits; although the precise functional significance of these mechanisms remains unknown (see also Wegesin et al., 2002; Li et al., 2004; for a critique of the compensation account see Friedman, 2013).

**Figure 2:**
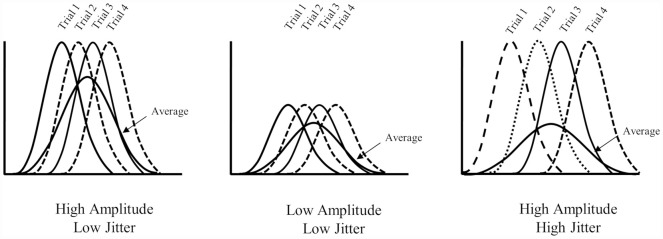
Illustration of the effect of different latency variabilities. The far left panel illustrates increased amplitude with low latency jitter, the middle panel shows low amplitude and low jitter and the far right illustrates increased amplitude and high jitter. Note that the average waveform in the far right panel mimics the average waveform observed in the middle panel (i.e., low amplitude).

In the present article, we attempt to address a specific problem for ERP investigations of older adult populations that may explain the inconsistent results between episodic recognition studies—namely, the issue with age-related increases in the latency variability of ERP components. To be clear, the level of engagement of a particular cognitive process is correlated with the amplitude of specific components of ERP waveforms averaged from a number of single trials with homogeneous cognitive process. It is assumed that the onset of a particular process is relatively consistent across single trials—although it is well known that this assumption is often violated (for discussion, see Spencer, 2005). When the latency of a particular component varies across trials (i.e., is subject to latency jitter), the averaged ERP can become blurred or smeared—depending on the affected condition(s)—and may obscure or mimic amplitude differences (see Figure 2). Schizophrenic patients, for example, demonstrate smaller P3 amplitudes than healthy controls (e.g., Jeon and Polich, 2003); however, the effect may be accounted for by different extents of latency jitter as schizophrenic patients often demonstrate larger reaction time variability (Ford et al., 1994). Similarly, reaction times also become more variable with age (MacDonald et al., 2008) and some ERP components such as the P3 have been shown to increase in latency among older adults (Polich et al., 1985; Polich, 1997; although see Walhovd et al., 2008). Amplitude variation in averaged ERP waveforms, across conditions or populations, therefore reflect a mixture of the true amplitude variation and different extents of latency jitter (Ouyang et al., 2016). If the latency jitter is significant across trials, true condition effects in amplitude can become suppressed (see Figure 2, far right)—weakening statistical analyses and making comparisons of ERP data between conditions or populations difficult to interpret. In short, current ERP aging studies are ambiguous as to whether age-related changes in magnitude of recollection related old/new effects reflect genuine changes in engagement of recollection (or at a minimum whether the effect is even observed) or simply greater trial-to-trial latency variability among older adults.

While the problem of latency jitter is well established (see Picton et al., 1984; Möucks et al., 1988; Spencer, 2005; Luck, 2014), effective solutions remain relatively under-explored (for a review, see Ouyang et al., 2016). Here, we employ a recent approach to compensating for component-specific trial-to-trial latency variability known as Residual Iteration Decomposition Analysis (RIDE: Ouyang et al., 2011, 2015a,b, 2016). Below, we briefly summarize the RIDE approach (a detailed account of the algorithm can be found in Ouyang et al., 2015a). RIDE assumes that ERPs consist of latency-invariant (e.g., early, stimulus-locked components) and latency-variant component clusters (e.g., late, response-related components). RIDE can decompose ERPs into different component clusters with different extents of latency variability. In principle, RIDE separates ERPs into stimulus-locked, response-locked and intermediate non-marker-locked component clusters. Components locked to either the stimulus or response are placed into “S” and “R” clusters, whilst components neither locked to stimulus or response are classified into the central (or intermediate) “C” cluster. The single-trial latencies of the intermediate component C can then be automatically estimated based on single-trial data. Whilst every waveform will include both S and C components, R components are not necessarily required—specifically in experimental designs whereby no response is made within the selected time window. In “response-free data” (when no overt response is involved, or where behavioral responses are made out-with the critical ERP time-window), the steps for estimating R in the decomposition module can be dismissed^2^.

**Figure 3:**
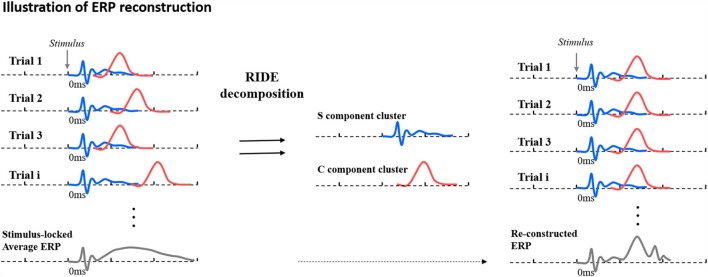
Schematic illustration of conventional stimulus-locked average Event Related Potential (ERP) (left) from single trials with latency jitter, decomposition byResidual Iteration Decomposition Analysis (RIDE; middle) and reconstructed ERP after correcting for latency variability (right). The S component is shown as a blue waveform and the C cluster as a red waveform. Figure is adapted from Ouyang et al. (2016).

After the decomposition of ERPs into different clusters, with different latency jitter relative to stimulus onset (for S, the latency jitter is zero), the average ERPs can be reconstructed—placing each component at the most probable latency and re-averaging them to form RIDE-adjusted ERPs (rERPs). A concise illustration of RIDE working is shown in Figure 3. In essence, RIDE can be used to reconstruct the jitter-compensated ERPs that disambiguate the confounding effects of amplitude variation and latency jitter on ERP amplitudes. RIDE has been consistently found to effectively recover amplitude effects that are diminished or exaggerated in conventional averages by trial-to-trial latency jitter (see Berchicci et al., 2016; Kashyap et al., 2016; Ouyang et al., 2016; Wolff et al., 2017; Bluschke et al., 2018; Bodmer et al., 2018). Although RIDE has yet to be used to test for amplitude changes among older adults, we predict that the algorithm will allow us to more accurately characterize retrieval related effects associated with recollection.

Here, we assess the neural correlates of recollection in older adults, using a similar continuous source paradigm employed by Murray et al. (2015). We operationalize recollection as reflecting the recovery of contextual information that was not present at test—in this case, an arbitrary location associated with a previously studied word. As with our earlier article (Murray et al., 2015), neural activity is measured as a function of the accuracy of source location responses. Electrophysiological data is first analyzed using traditional averaging techniques and followed up with analysis of rERPs. The continuous source task allows us to make explicit predictions about pattern of retrieval success effects we expect to observe depending on how recollection operates. First, we expect to see a clear effect of RIDE, with more robust retrieval success effects present after the adjustment (explaining why many previous ERP studies of aging and memory report no effects). Second, the pattern of retrieval success effects observed will reflect how recollection operates. According to the some-or-none account of recollection the neural correlate of recollection seen in older adults will be sensitive to accuracy in much the same way as in younger adults—i.e., absent for Guesses, but increasing in magnitude with accuracy when trials exceed a threshold. Alternatively, if recollection operates in an all-or-none fashion then retrieval success effects will be absent for guesses but will be present and equal in magnitude for all trials that exceed a threshold. As we show below, whilst the traditional ERPs suggest very weak and statistically marginal retrieval success effects, the rERPs reveal a more robust set of retrieval success effects that arbitrates between the different accounts of how recollection operates in older adults.

## Materials and Methods

### Participants

Twenty-four participants of whom 12 were female (mean age = 68, range = 60–78 years) gave informed consent (approved by the University of Stirling Psychology Ethics Committee). Participants were recruited from the general Stirling community. Before the experiment, participants were required to undergo a series of assessments and questionnaires to prevent the inclusion of atypical participants, including self-rating of general health (one being poor and six being excellent), forward and backward digit span tasks (Wechsler, 1981), the 15-item Geriatric Depression Scale (GDS-15; Sheikh and Yesavage, 1986) and the Mini-Mental State Examination (MMSE; Folstein et al., 1975). The results of these assessments are reported in Table 1. We also ensured that every participant had normal or corrected-to-normal vision as measured using Snellen Eye Chart [where scores range from 1 (20/200 visual acuity) to 11 (20/10 visual acuity)]—again, the results are reported in Table 1. It is important to note that although we were able to ensure sufficient visual acuity in all our participants, we cannot rule out the possibility that variation in eye sight may have resulted in subtle effects on memory performance. Three participants decided to end the experiment prematurely and were excluded from the final analysis. Reported below are the data from the remaining 21 participants.

**Table 1:** Mean and Standard Deviations (SD) for Older adults with regards to years of formal education, general health, forward and backward digit span, visual acuity and Geriatric Depression Scale (GDS) scores.

Assessments	Older adults (*N* = 21)
Years of education	11 (3)
General health	5 (1)
Forward digit span*	10 (2)
Backward digit span**	9 (2)
MMSE***	29 (1)
Visual acuity	7 (1)
Geriatric depression scale	1 (2)

*Consistent with the average forward digit span test score of 9.9 (2.8; Ruchinskas, 2019). **Consistent with the average backward digit span test score of 10.2 (2.4; Ruchinskas, 2019). ***Slightly above the average MMSE score of 26.1 (1.1) reported by Ciesielska et al. (2016).

### Stimuli

Stimuli were identical to those employed by Harlow and Donaldson (2013) and Murray et al. (2015). Two-hundred and forty words were selected from the MRC psycholinguistic database (Coltheart, 1981)^3^. Words (examples include bother, pardon, ignore and remedy) shared similar length (between 5–7 letters) and were selected with low imagability (mean = 414, *SD* = 55, range = 235–500), concreteness (mean = 351, *SD* = 63, range = 222–498) and Kucera Francis word frequency (mean = 33, *SD* = 31, range = 1–142). Nine additional words were used for the practice block.

### Procedure

The experiment was conducted on a desktop PC, using E-Prime software (Version 1: Psychology software tools) and a standard keyboard and mouse. Words were presented in black on a white background, using lower case 18 point Courier New font: the gray circle outline and black location cross were also presented on a white background. Participants were given both verbal and written instructions explaining the task and response demands. Prior to the main experiment, participants were required to complete a short practice (using nine words/location pairs). The main experiment involved 12 study blocks of 20 words/location pairs and 12 test blocks (again consisting of 20 words/location pairs), with each study block followed by the corresponding test block.

Participants pressed the space bar to begin each study trial (see Figure 1A) and were presented with a black cross located on a gray circle outline (600 ms), followed by a 250 ms blank screen and then a word for 1,500 ms. To ensure that participants had successfully encoded the correct location, they were asked to verify the (now hidden) location. Participant’s attention to the location was then tested by asking them to verify the (now hidden) location using the mouse. Responses within 20 pixels (~6°) from the target advanced participants to the next trial. If the participant’s response was over 20 pixels, the target location was presented again (250 ms) and the verification task was repeated.

During every test trial (see Figure 1B), participants were presented with a fixation cross (1,000 ms) followed by blank screen (500 ms). A word from the previous study block was then shown (1,000 ms), followed by a blank screen (1,000 ms). Participants were then presented with a gray circle outline and were asked to recall the paired location by moving the mouse cursor to the remembered location and clicking the left mouse button. A red marker then appeared on the circle to indicate the chosen location. No response time limit was set and participants were free to change their decision^4^. Participants finalized their response by pressing the space bar to initiate the next trial. After the experiment was complete the accuracy of each test response was calculated, converting screen co-ordinates selected by the participant into degrees: the remembered location was compared to the target location to provide the degree of error on each test trial (see Figure 1C).

### Event Related Potential Data Acquisition and Processing

EEG was measured at the scalp using 62 silver/silver chloride electrodes embedded in an elasticated cap (Neuromedical supplies: www.neuro.com) in accordance with an extended version of the International 10/20 system: (FP1, FPZ, FP2, AF3, AF4, F7, F5, F3, F1, FZ, F2, F4, F6, F8, FT7, FC5, FC3, FC1, FCZ, FC2, FC4, FC6, FT8, T7, C5, C3, C1, CZ, C2, C4, C6, T8, TP7, CP5, CP3, CP1, CPZ, CP2, CP4, CP6, TP8, P7, P5, P3, P1, PZ, P2, P4, P6, P8, PO7, PO5, PO3, POZ, PO4, PO6, PO8, CB1, O1, OZ, O2, CB2; see Figure 7, inset). The ground electrode GND was positioned midway between AF3 and AF4. During recording, each electrode was referenced to an additional electrode midway between CZ and CPZ. All channels were re-referenced offline to a virtual mastoid that was calculated by averaging the signal from electrodes located on the left and right mastoids. Vertical and horizontal EOG was recorded from bipolar pairs of electrodes placed above and below the left eye, and on the outer canthi. Electrode impendence was kept below 2 kΩ. EEG and EOG data was amplified with a band pass filter of 0.1–40 Hz, digitized by a 16-bit analog to digital converter at a sampling rate of 250 Hz and recorded on a desktop computer using NeuroScan Aquire software (version 4.3). EEG data were processed using NeuroScan Edit (version 4.3).

**Figure 4:**
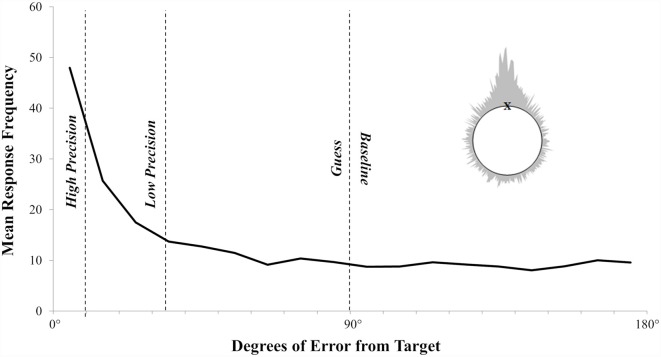
The observed error distribution clearly shows a mixture of thresholded recollection with responses clustering around the target and sub-thresholded guessing that is uniformly distributed above zero. The dashed lines represent the division of ERP bins. Inset is the wrapped distribution of errors from both the left and right side of the target.

**Figure 5:**
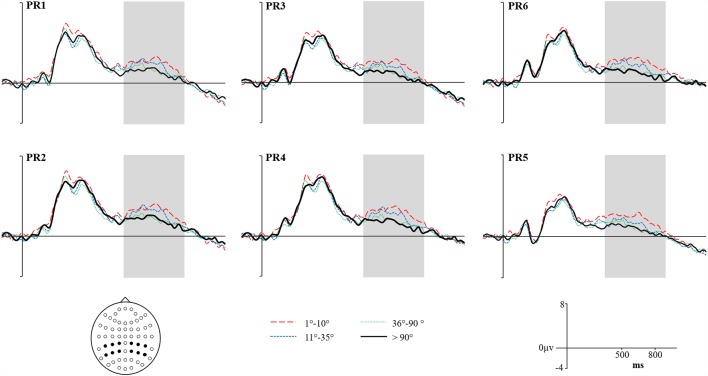
Grand average ERPs for High Accuracy responses (1°–10°) shown as a red dashed line, Low Accuracy responses (11°–35°) shown as a blue dashed line, Guess responses (36°–90°) shown as a Green dotted line, and Baseline (over 90°)—shown as a solid black line. Representative electrodes are shown over the left Parietal Region (PR; top row: PR6, PR3, PR1) and right PR (bottom row: PR6, PR4, PR2). Inset is the schematic map of the 62 recording electrodes—with those representative electrodes represented as black dots.

**Figure 6:**
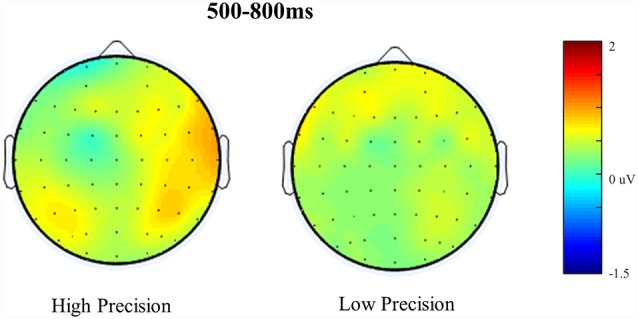
Scalp maps illustrating old/baseline distributions for High Accuracy and Low Accuracy responses during the 500–800 ms time window. Each map is shown as if looking down on the head with frontal sites pointing towards the top of the page. The scale bar indicates the voltage range (μV).

**Figure 7:**
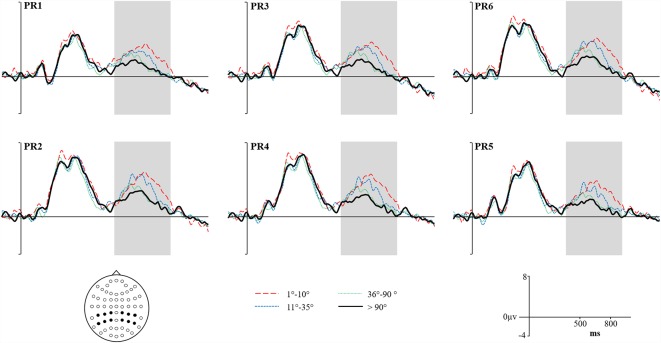
Grand average Reconstructed ERPs for High Accuracy responses (1°–10°) shown as a red dashed line, Low Accuracy responses (11°–35°) shown as a blue dashed line, Guess responses (36°–90°) shown as a Green dotted line and Baseline (over 90°)—shown as a solid black line. Representative electrodes are shown over the left PR (top row: PR5, PR3, PR1) and right PR (bottom row: PR6, PR4, PR2). Inset is the schematic map of the 62 recording electrodes—with those representative electrodes represented as black dots.

Raw EEG data were inspected and any segments including high levels of noise (i.e., artifacts including excessive muscle movements) were removed. An ocular artifact reduction procedure was applied to reduce the effects of eye blinks: 32 optimal blinks from each participant were selected to estimate the individuals blink pattern and remove the contribution of the average blink from all channels. The EEG data was then epoched and time-locked to stimulus presentation at test, using a 2,040 ms time window (starting with a −104 ms pre-stimulus baseline). Epochs were rejected if they had a drift from baseline exceeding ±75 μV, or where the signal change exceeded ±100 μV. ERPs were then formed by averaging together all trials within a series of response accuracy bins (described in more detail in the “Results” section); the data was then baseline corrected and smoothed with a 5-point kernel. For the initial analysis at least 16 artifact-free trials were required from each participant, in each of the critical response bins to ensure a good signal-to-noise ratio.

### Residual Iteration Decomposition Analysis (RIDE)

The RIDE analysis was performed on the processed EEG data imported from Neuroscan and closely follows the method described in detail by Ouyang et al. (2015a). RIDE was applied using the MATLAB toolbox package and manual available on http://cns.hkbu.edu.hk/RIDE.htm. Below we provide an overview of the general procedure. At first, RIDE identifies the single-trial latencies of the both the S and C clusters i.e., ERP = S + C. The latency of S is taken from the stimulus onset marker and the latency of C is estimated by template matching between template and single trials. The template is initially taken from the average ERP and later replaced with C component after the first iteration of decomposition. Given the two latency sets, RIDE employs an iterative decomposition scheme to isolate both clusters based on the procedure as follows: to obtain a single component cluster (e.g., S), RIDE subtracts all the other component clusters from each single trial, aligns the residual of all trials to the latency of the component cluster and obtains the waveform as the median waveform over all time points. The same procedure is applied to obtain each component cluster iteratively till convergence. The median waveform was used to immunize from noise amplification from iteration (Ouyang et al., 2015b), but was restored to the average waveform in the end, in line with standard ERP. The decomposition procedure is re-done after updating of C latency estimation until the convergence of C latency estimates, which form an outer iteration.

As mentioned above, rERPs can be constructed after RIDE, allowing for the analysis of grand average ERPs that represents a waveform corrected for potential smearing and is most representative of single trials. The rERPs were obtained by placing each component cluster at single trial after re-synchronizing them to the median of their latencies (illustrated in Figure 3) and averaging the re-synchronized single trials. More details concerning the algorithms applied in the current study can be found in Ouyang et al. (2015b).

### Analysis Strategy

ERPs were analyzed by examining mean amplitudes (relative to the pre-stimulus baseline) during *a priori* defined time-window, designed to capture the neural correlate of recollection (500–800 ms). To allow retrieval success effects to be calculated, all average ERPs were compared to the Baseline ERP, constructed from trials associated with responses over 90° from the true target location. The baseline was chosen because error responses in the opposite half of the circle from the target are not based on recollection, providing an ERP baseline that is analogous to a missed condition during standard old/new analysis (identical to the method employed by Murray et al., 2015). All ERP data analysis was carried out using repeated measures ANOVA (specific factors and levels are described where appropriate in the “Results” section). The Greenhouse-Geisser correction for non-sphericity was applied and adjusted degrees of freedom are reported when necessary. Once significant effects were found, topographic analyses were carried out on subtraction waveforms; these data were re-scaled using the min/max method (McCarthy and Wood, 1985) to avoid the potential confound of differences in the size of effects.

ERPs were formed for each participant, averaging EEG data recorded at test into four separate response categories. To facilitate comparison with previous studies the response categories were based on those employed by Murray et al. (2015), designed to capture ERP activity elicited by different rates of positional response accuracy, whilst also providing sufficient trial numbers to form ERPs across participants. High Accuracy’ ERPs were designed to capture only the most precise responses, defined as under 10° from the target location; “Low Accuracy” ERPs captured less precise responses, ranging from 11° to 35° from target; “Guesses” ERPs captured trials associated with guessing, ranging from 36° to 90°. Critically, the Guesses ERPs reflect responses in the same half of the circle as the target, but from a part of the distribution largely accounted for by the plateau of random guessing. Finally, “Baseline” ERPs were formed from all trials falling over 90° from the target location (i.e., the other half of the circle, analogous to wrong answers in a binary source task), well beyond the guessing asymptote suggested by the analysis of individual participant data. The mapping of ERP response category to behavioral data is illustrated in Figure 4. Although the range and number of response categories are essentially arbitrary, the set of response bins was chosen to allow us to form robust ERPs across all participants, whilst also effectively capturing variability in the accuracy of responses. The mean number of trials contributing to each response bin were as follows: High Accuracy (mean = 44, *SD* = 19, range = 20–21), Low Accuracy (mean = 45, *SD* = 11, range = 31–78), Guess (mean = 55, *SD* = 11, range = 29–72) and Baseline (mean = 75, *SD* = 19, range = 35–103).

## Results

### Behavioral Data

To ensure that stimuli were sufficiently attended to at encoding, participants had to verify each location presented at study. As expected, participants were highly accurate at verifying the target location: 93% (*SD* = 0.05, range = 87%–98%) of participant’s initial responses were within 10° of the target. The observed proportion of errors at encoding was very similar to the 92% of responses in younger adults observed by Murray et al. (2015). In comparison to the encoding data, only 20% of older adult’s responses at retrieval were within 10°—revealing a far lower level of accuracy.

The overall pattern of test responses is shown in Figure 4. As with the error distributions observed elsewhere (see Harlow and Donaldson, 2013; Murray et al., 2015; Harlow and Yonelinas, 2016), the observed responses here show a mixture of variable responses that cluster around the target and a random distribution of guessed responses. The data were analyzed using the modeling procedures introduced by Harlow and Donaldson (2013). To be clear, to examine whether responses made at test exhibit a thresholded or continuous pattern, the error distribution for each participant is fitted to a threshold model of recollection, with two free parameters. A threshold parameter, λ, denotes the proportion of trials on which recollection succeeds. Errors on these recollected trials follow a wrapped Cauchy distribution with shape parameter *s*, denoting the spread of responses around the target with higher values of *s* indicating a greater mean error (i.e., lower accuracy). The remaining 1 − λ non-recollected trials are guesses, randomly distributed around the circle relative to the target, resulting in a uniform distribution of errors (see Figure 4).

On average, older participants recollected 66% (λ) of the locations, and the mean recollected trial was 15.39° (*s*) from the target. To decide whether recollection in older adults is best described as a threshold or a continuous process, we compared our model by either fixing the value of λ at 1 (such that all responses are based on some variable amount of recollection, consistent with a continuous account), or allowing λ to vary below 1 (such that recollection could fail on a subset of responses, consistent with a threshold account, and resulting in random guessing around the circle). To detect the existence of a threshold we conducted a likelihood ratio test. By allowing λ to vary below 1 we significantly improved the likelihood of the observed data in older adults compared to fixing λ at 1 [mean *λ* = 0.66 (21), *χ*^2^ = 602.09, *p* < 0.001]. Consistent with previous findings with younger adults (i.e., Harlow and Donaldson, 2013; Murray et al., 2015; Harlow and Yonelinas, 2016), our data strongly suggests that the distribution of error responses for older adults is also accurately modelled with a threshold rather than a continuous distribution.

Response time data was also analyzed for each response category. A series of bonferroni corrected *t*-tests (corrected *α* = 0.01) revealed that High Accuracy trials (mean = 1730 ms, *SD* = 683 ms) were associated with significantly quicker response times than both Low Accuracy trials (mean = 2,153 ms, *SD* = 1,179 ms); (*t*_(1,21)_ = 3.41, *p* < 0.001, *d* = 0.73), Guess trials (mean = 2,497 ms, *SD* = 1,378 ms); (*t*_(1,21)_ = 4.43, *p* < 0.001, *d* = 0.95), and Baseline trials (mean = 2,623 ms, *SD* = 1,524 ms); (*t*_(1,21)_ = 4.34, *p* < 0.001, *d* = 0.93). In addition, Low Accuracy trials were also significantly quicker than either Guess trials (*t*_(1,21)_ = 3.94, *p* = 0.001, *d* = 0.84), and Baseline trials (*t*_(1,21)_ = 3.01, *p* < 0.01, *d* = 0.66). Comparison of Guess and Baseline trials, however, did not reveal any significant difference in response times (*t*_(1,21)_ = 1.07, *p* = 0.3, *d* = 0.23).

We also examined the individual variability in responding, to assess the consistency of the response threshold across participants. Importantly, the pattern of responses observed in the majority of participants is indicative of a threshold—matching the average data. For a small number of participants, the pattern becomes less clear with some exhibiting very precise responding and others far greater guessing (with a flatter profile of responses around the circle). Regardless, analysis revealed that 19 out of 21 participants demonstrate a threshold when analyzed individually. In addition, the values of λ and s differed considerably across participants (with values ranging from 0.53 to 0.84, and 7 to 22, respectively) reflecting the variation in response profiles across participants.

### The Parietal Retrieval Success Effect

Figure 5 shows the Grand Average ERPs for High, Low, Guess and Baseline responses at representative electrodes. Although there is no clear separation, High accuracy responses appear to be slightly more positive going than Low accuracy responses, which are both slightly more positive going than either the Guess or Baseline responses (which do not differ). The topographical distribution of the retrieval success effects is illustrated in Figure 6: only High accuracy responses appear to elicit clear parietal effects, with greater magnitude over the right than left hemisphere. By comparison, the effects seen for Low accuracy responses appear very weak, with a slight frontal and right parietal distribution. The experimental prediction that the parietal retrieval success effect would be sensitive to accuracy was tested by averaging across Parietal and Centro-Parietal electrodes {identical to the ERP analysis strategy employed by Murray et al., 2015; CP5/P5 [Parietal Region (PR)5], CP3/P3 [PR3], CP1/P1 [PR1], CP2/P2 [PR2], CP4/P4 [PR4], CP6/P6 [PR6]} and comparing activity elicited for correctly recollected, guessed and baseline responses. Initial ANOVAs were performed on each response category to test for within category retrieval success effects, with factors Category (High, Low or Guess vs. Baseline), Hemisphere (Left vs. Right) and Site (Inferior vs. Middle vs. Superior) during the 500–800 ms time window.

ANVOAs with factors of Category (High, Low or Guess/Baseline), Hemisphere (Left/Right) and Site (Inferior/Middle/Superior) comparing each response category to Baseline revealed a marginally non-significant main effect of Category for High accuracy responses (*F*_(1,20)_ = 3.373, *p* = 0.07, $\eta_{\text{p}}^{2}$ = 0.15), a significant interaction between Category and Site (*F*_(1.22,24.45)_ = 8.87, *p* < 0.001, $\eta_{\text{p}}^{2}$ = 0.31) and marginally non-significant Category, Hemisphere and Site (*F*_(1.23,24.49)_ = 3.50, *p* = 0.07, $\eta_{\text{p}}^{2}$ = 0.15) interaction. The weak effects observed for High Accuracy responses appear to be driven by greater differences over inferior parietal electrodes. Although no significant interaction with Hemisphere was observed, the difference between High Accuracy and Baseline responses was numerically larger over the right [mean μV at PR6 = 1.16, (*SD* = 2.16)] compared to the left hemisphere [mean μV at PR5 = 0.75, (*SD* = 1.02)]. No significant main effects or interactions were observed for either Low accuracy or Guess responses.

The pattern of ERP retrieval success effects reported above suggests that, for older adults, parietal effects are marginal for High Accuracy responses but absent for Low Accuracy responses—suggesting that the recollection operates differently compared to younger adults (whereby significant parietal effects were observed for both high and low accuracy trials). It is important to note, however, that although no significant effects are observed for Low Accuracy responses the waveforms shown in Figure 5 do appear to be marginally more positive than either Guesses or the Baseline. As suggested in the introduction, one possibility for the weak effects observed among older adults could be greater latency variability that may cause smearing in the averaged waveform. In the section reported below, we apply RIDE to correct the observed effects for both High and Low Accuracy responses that are likely to be attenuated by latency jitter.

### RIDE Corrected Waveforms

**Figure 8:**
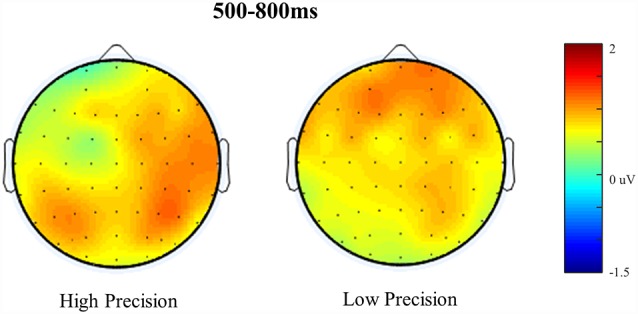
Scalp maps illustrating Reconstructed ERP old/baseline distributions for High Accuracy and Low Accuracy responses during the 500–800 ms time window. Each map is shown as if looking down on the head with frontal sites pointing towards the top of the page. The scale bar indicates the voltage range (μV).

We report the reanalyzed RIDE corrected data with the same analysis strategy employed on the original uncorrected data. The Reconstructed ERPs are shown in Figure 7. It is clear that the waveforms from the representative electrodes show a much more defined morphology, with both High and Low accuracy responses being more positive than either Guesses or Baseline. Similarly, the topographical distribution of the retrieval success effects is illustrated in Figure 8. The topographic maps reveal much clearer retrieval success effects across anterior and posterior electrodes (compared to those visible in Figure 8). Critically, the parietal retrieval success effects are now visible for both the High and Low Accuracy response categories, with a bias towards larger effects over the right than left hemisphere in both cases.

An ANOVA with factors of Category (High Accuracy, Low Accuracy, Guess/Baseline), Hemisphere (Left/Right) and Site (Inferior/Middle/Superior) revealed significant main effect of Category for High (*F*_(1,20)_ = 6.45, *p* < 0.05, $\eta_{\text{p}}^{2}$ = 0.24) and Low (*F*_(1,20)_ = 4.32, *p* = 0.05, $\eta_{\text{p}}^{2}$ = 0.18) Accuracy responses. Analysis of the RIDE corrected High Accuracy responses also revealed a Category by Site (*F*_(1.23,24.56)_ = 8.21, *p* = 0.01, $\eta_{\text{p}}^{2}$ = 0.29) interaction and a three-way Category, Hemisphere and Site (*F*_(1.2,23.9)_ = 5.04, *p* < 0.05, $\eta_{\text{p}}^{2}$ = 0.20) interaction. Closer inspection of each hemisphere revealed a significant Category by Site interaction over the Right Hemisphere (*F*_(1.24,24.79)_ = 9.93, *p* < 0.01, $\eta_{\text{p}}^{2}$ = 0.33) but not Left Hemisphere (*p* = 0.38, $\eta_{\text{p}}^{2}$ = 0.04), suggesting that the retrieval success effect was largest over inferior electrodes, with a right greater than left asymmetry. No further interactions were observed for Low Accuracy trials, although the effect was numerically largest over electrode PR4 (mean μV = 0.88, *SD* = 1.75). Lastly, no significant main effects or interactions were observed for Guess responses.

Having demonstrated that retrieval success effects were present, a topographic analysis was carried out to assess whether the effects observed for High and Low Accuracy responses could have been generated by different neural populations. An ANOVA was performed using rescaled subtraction data (High Accuracy-Baseline/Low Accuracy-Baseline) with factors of Category (High/Low) and Site (62) was carried out, thereby providing a global assessment of the topography of effects across the scalp. These data revealed no evidence of a Category by Site interaction, indicating that the two scalp distributions are statistically equivalent.

To directly compare the magnitude of Parietal retrieval success effects we submitted the unscaled subtraction data to an ANOVA with factors of Condition (High/Low), Hemisphere (Left/Right) and Site (Inferior/Middle/Superior). Analysis revealed both a Condition by Site interaction (*F*_(1,20)_ = 10.03, *p* < 0.01, $\eta_{\text{p}}^{2}$ = 0.33) and a significant three-way Condition, Hemisphere by Site interaction (*F*_(1,20)_ = 7.25, *p* < 0.01, $\eta_{\text{p}}^{2}$ = 0.26). Next, we further explored this three-way interaction by carrying out a Condition by Site analysis for both the left and right hemispheres. Across the Left Hemisphere, our results revealed no significant main effect of Condition, nor any significant interaction (*p* = 0.84, $\eta_{\text{p}}^{2}$ = 0.04). By contrast, analysis of the Right Hemisphere revealed a significant Condition by Site interaction (*F*_(1.34,26.8)_ = 17.22, *p* < 0.01, $\eta_{\text{p}}^{2}$ = 0.46), reflecting the fact that the differences between High than Low accuracy ERPs are larger at inferior than superior electrodes, however, no significant main effect of Condition was observed.

Despite demonstrating a difference in the pattern of parietal effects between conditions, our analysis technique did not detect any main effect of Condition. These results are therefore not as compelling as those found in our previous article (Murray et al., 2015) focusing on the same parietal retrieval effects in younger adults—whereby direct comparisons of the magnitude parietal retrieval success effect were observed between High and Low Accuracy conditions. Previously, however, we also ran a subsidiary analysis using more fine-grained bins (ranging from 1° to 90° of error) to explore the correlation between the size of the parietal effect and response accuracy. Here, we carry out a similar analysis to explore the strength of relationship between parietal activity and response accuracy in older adults, using regression analysis to examine the pattern of effects in each hemisphere. To be clear, if there is a relationship between the size of the parietal effect and response accuracy we should expect to observe that the magnitude of the effect decreases as participants become less accurate.

Each regression was carried out on average of electrodes from the Left Hemisphere [PR5, PR3, PR1] and Right Hemisphere [PR2, PR4, PR6]. Identical to the analysis strategy carried out by Murray et al. (2015), first we created a series of response bins (every 10° of error) and averaged every trial within participants that fell within these bins, before averaging across all participants. As we were interested in the gradation of the neural signature of successful retrieval, we subtracted averaged activity from Baseline responses (i.e., 90° to 180°) from each bin. We employed weighted least squares regression, which assigns weights that are inversely proportional to the error variance of each bin—i.e., more accurate bins are given greater weight in the regression than inaccurate bins which exhibit greater variance. Analysis revealed a significant negative correlation between the magnitude of the Parietal Retrieval success effect and degree of error from target location across both the left hemisphere (*r* = 77, *p* = 0.02) and right hemisphere (*r* = 0.69, *p* < 0.05). Across the left hemisphere, response accuracy accounted for 59% of variance in the size of the magnitude of the parietal effect, and 47% of variance across the right hemisphere.

## General Discussion

In the present study, we used a continuous source task to assess whether the neural mechanism of successful episodic recollection was sensitive to retrieval accuracy in older adults. We were motivated by recent ERP evidence demonstrating that the magnitude of the neural mechanism supporting recollection (the left parietal effect) was sensitive to retrieval accuracy in younger adults. Here, we investigated whether the neural correlate associated with recollection success in older adults was also sensitive to retrieval accuracy. Although analysis of the traditional ERPs revealed only a weak and statistically marginal effect for High accuracy responses, the reconstructed ERPs obtained after RIDE revealed a much clearer pattern of results—i.e., the parietal effect was absent when recollection failed but was sensitive to the accuracy of recollected information when successful. The pattern of neural data observed in older adults appears to be consistent with the pattern of left parietal effects observed in younger adults, reflecting the operation of a thresholded and variable signal. Below, we discuss the implications of our findings for theories of age-related memory decline and functional accounts of the neural mechanism supporting successful episodic recollection.

Behaviorally, our data demonstrate that recollection in older adults’ functions as a some-or-none process—similar to the way recollection operates in younger adults. Specifically, recollection exhibits a threshold and sometimes fails completely, but is variable when successful. Although direct statistical comparisons cannot be made across studies, it is noteworthy that the rate of recollection observed in older adults is lower, on average, than the rate previously reported in younger adults (*λ* = 0.66 compared to 0.73 observed by Murray et al., 2015). In addition, the retrieval rate observed in older adults’ confirms that they were performing the task well above chance and below ceiling. Furthermore, the results also suggest a lower level of accuracy than observed in younger adults (*s* = 15.39° compared to 11.40° reported by Murray et al., 2015). Although we do not directly compare rate and accuracy between age-groups in the present study because we only tested older adults, the reduction in accuracy as a consequence of healthy aging is consistent with the conclusions of Nilakantan et al. (2018). Overall, the data supports the view that recollection, at a behavioral level of analysis, is best characterized as a some-or-none process in older age.

Our central question here, however, was whether the neural mechanism supporting recollection was sensitive to retrieval accuracy and exhibits a thresholded and variable pattern among older adults. We were able to investigate the neural correlate of recollection by comparing the magnitude of retrieval success effects as a function of positional response error. Several features of the data are important. First, parietal effects were found to be reliable only after correction for single-trial latency variability via RIDE. Initial analysis of the ERP data using standard processing procedures revealed a weak and a marginally non-significant parietal effect for High accuracy responses. We hypothesized, however, that the observed amplitudes in older adults could be affected by smearing, due to latency variability across single trials. Indeed, after correction of single-trial latency variability, we were able to observe a thresholded and variable pattern of ERP data—consistent with the behavioral evidence that successful memory performance relied on recollection. Second, the pattern of parietal retrieval success effects was thresholded: i.e., the effect was absent when recollection failed (as indexed by guess responses with errors between 36° and 90°), but the magnitude of the effect correlated with accuracy when recollection was successful. To be clear, the magnitude of the parietal retrieval success effect scaled with response accuracy, such that higher accuracy responses were associated with larger ERP effects than lower accuracy responses. Finally, for High Accuracy responses (i.e., 1° to 10°), the parietal effect was localized over inferior electrode sites over the right as opposed to left hemisphere—a topography that is inconsistent with the neural correlate of recollection in younger adults but in line with previous ERP source memory studies of older adults (see Wegesin et al., 2002; Li et al., 2004; Duverne et al., 2009). Overall, the novel finding that parietal activity in older adults is sensitive to response accuracy provides further evidence that the effect is associated with recollection in older adults (see also Li et al., 2004).

### Residual Iteration Decomposition

**Figure 9:**
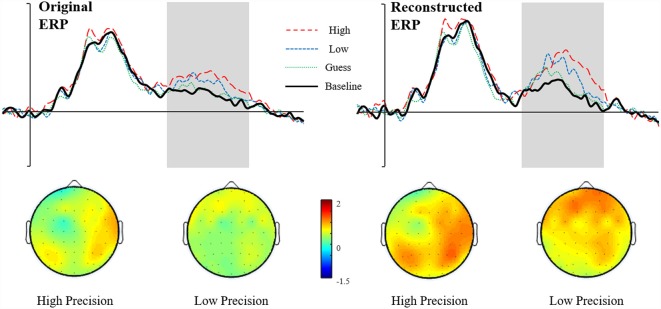
Figure illustrating the effect of RIDE: on the left is the original ERP data (waveforms shown at representative electrode PR4) with the topographies for High and Low Accuracy responses directly below. On the right side is the Reconstructed ERP data (waveforms shown at electrode P5) with topographies for High and Low Accuracy shown below.

Although the pattern of results supports the view that recollection is thresholded in older adults, our conclusions ultimately depend upon the interpretation of the reconstructed averaged ERP *via* RIDE—an established algorithm for correcting latency variability that can obscure the analysis of ERP amplitudes. In principle, the reconstructed ERP should exhibit enhanced amplitudes, with a more clearly defined structure as a result of the de-blurring by correction of trial-to-trial latency jitter. In practice, this improvement in data quality is exactly what was observed in our data. As can be seen in Figure 9, the marginal effects observed in the original ERP data (Figure 9, left side) were enhanced, revealing a much clearer pattern of results (Figure 9, right side). Our novel application of RIDE to reassess ERPs from older adult populations highlights the importance of checking for potential trial-to-trial latency variability (particularly when analyzing and comparing between different populations).

Our use of RIDE was used based on *a priori* concerns about the pattern of parietal effects within the ageing and memory literature. We would, however, warn against using RIDE *post hoc* to artificially enhance already observable effects or to detect effects that are simply not present to begin with (in fact RIDE will not produce significant effects if such effects were not at least observable in the original ERP data as demonstrated in Ouyang et al., 2016). In addition, there are, as with any processing method, certain limitations of RIDE that must be considered. First, because the method involves estimating the latency of the C component by cross-correlation, RIDE is sensitive to levels of background noise and signal-to-noise ratio. Specifically, latency estimation can be significantly affected by large artifacts which must first be identified and removed before separation. Furthermore, reliable estimation of the C component shape can only be achieved if there is a sufficient signal-to-noise ratio. Previous stress testing of the algorithm suggests that RIDE can effectively separate robust and reliable components using between 30 and 40 trials per condition (Ouyang et al., 2015b). Second, RIDE assumes that there are at least three component clusters (the stimulus-locked cluster, the response-locked cluster, and the cluster neither associated with the stimulus or response). Obviously, this is an over-simplification of the typical structure of the ERP because the C cluster is often more heterogeneous than is assumed by RIDE. Separating more than one component within the C cluster is challenging because the morphology of each cluster must be independent from one another (in that estimating single-trial latencies of one component will not affect the other). Consequently, some components are more difficult to separate than others, with the method working best on large, late endogenous components such as the P3, N400 and P600, whilst early components such as the P2, N2 or P1 are more difficult to separate. In general, RIDE is a powerful tool for correcting single-trial latency variability so long as the experiment is designed well, the appropriate components for analysis are selected and the data is of sufficient quality.

### Theoretical Implications

From a theoretical perspective, our results help clarify the functional significance of the parietal retrieval success effect in older adults. As noted in the introduction, although there is significant evidence associating recollection with the left parietal effect in younger adults, the relationship between parietal activity (whether maximal over the left or right hemisphere) and recollection in older adults is less consistent. Whilst some studies fail to observe any parietal activity at all, other studies appear to show a more right-sided topography (Wegesin et al., 2002; Li et al., 2004; Duverne et al., 2009). Here, our results demonstrate that the parietal effect was largest at inferior sites across the right hemisphere, but did not reveal an overall difference in parietal magnitude between hemispheres. In addition, our regression analysis revealed that parietal activity was sensitive to response accuracy across both hemispheres making it difficult to conclude that our effect was strongly right-lateralized. Regardless, our novel results help provide further evidence in support of the view that parietal activity observed in older adults’ correlates with recollection based retrieval by demonstrating that the ERP effect is sensitive to the quality of remembered information.

One limitation of the current study is that we did not directly compare the magnitude of retrieval effects between younger and older adults. Our data, therefore, cannot address whether there are age-related changes to size of the parietal effect. However, the aim of the study was to directly investigate the nature of recollection in older adults, not to address age-related changes in the accuracy of recollection. We believe that any significant age-related change in neural activity is difficult to interpret for a number of reasons. First, it is difficult to separate age-related effects from task-related effects given a significant behavioral difference in performance. To be clear, any significant reduction of the parietal effect as a function of age might simply reflect that, on average, older adults perform more poorly on a given task than younger adults. Second, it is relatively difficult to control for cohort effects whereby any age-related change in behavior, or neural activity, may simply reflect the time in which someone was born rather than their age. Cohort effects, in particular, have been shown to significantly influence both episodic recognition and recall (Rönnlund and Nilsson, 2009; for a more in depth discussion, see Rugg, 2016) and are extremely difficult to control. Lastly, cross-sectional ageing studies are generally difficult to interpret, particularly in ERP data, given that the size of the parietal retrieval success effect does not correlate with behavioral performance across individuals. MacLeod and Donaldson (2017), for instance, found that within two large samples of healthy young adults, neither subjective nor objective estimates of recollection correlated with the size of the Left Parietal effect between individuals, suggesting that differences in the magnitude of old/new effects found between groups (including younger and older adults) cannot be used to infer differences in recollection. Thus, our view is that the only definitive way of assessing whether there are age-related reductions in the magnitude of parietal effects is through longitudinal designs assessing the changes in cognitive function within individuals over an extended period of time.

### Future Directions

The data does, however, demonstrate that it is possible to capture variability in the accuracy of retrieval in older adults using both behavioral and electrophysiological methods. Our data also supports the view that a full account of cognitive ageing must consider variation in both the rate and accuracy of successfully remembered information. The concept of retrieval accuracy is only useful if it can be experimentally separated from retrieval rate via a double dissociation, however, as of yet, no such dissociation has been experimentally demonstrated. For instance, it is theoretically possible that one may have lower rates of retrieval but when successful remember with greater accuracy and by comparison, remember frequently but with poorer accuracy. Consistent with this view, Richter et al. (2016) demonstrated that both the rate and accuracy of recollection may rely on different brain regions related to a core recollection network. In that study, fMRI data suggested that the rate of recollection correlated with activity in the hippocampus, whereas the angular gyrus was sensitive to recollection accuracy. Given this possible neural dissociation, it therefore entirely possible that decreases in retrieval rate may not necessarily predict a decrease in accuracy. Clearly, more evidence is required attempting to tease apart the rate and accuracy dimensions of recollection tested using a broad range of experimental measures.

If the rate and accuracy were shown to be functionally separable, however, then understanding retrieval accuracy becomes critical to our understanding of age-related memory decline. Variation in memory quality, for example, arguably provides a more sensitive measure of retrieval success than simply measuring whether an older adult can successfully remember an event. In practice, the ability to detect subtle changes to memory accuracy, which may become apparent before memory failure, could aid in the early detection of abnormal ageing caused by disease such as dementia. It is also currently unclear whether the properties of a stimulus or particular encoding strategy may have a differential impact on retrieval accuracy that is not evident from traditional measures of retrieval rate. Such findings would be critical for the development of behavioral interventions designed for those with selective recollection deficits who may benefit from enhanced retrieval accuracy.

Our data also has specific implications for ERP analysis of older adults. As we have made clear, differences in the grand average ERP amplitude between conditions or groups may reflect genuine changes in the cognitive processes under investigation or simply variation in the latency variability of single trials. Indeed, while latency variability in different groups of participants is well known, the problem is often ignored in studies that interpret averaged ERPs in older populations. We hypothesized that potential latency variability may be one important factor contributing to the inconsistent pattern of retrieval-related parietal effects within the aging literature. As different populations of older adults will be used across studies, it is possible that different datasets will vary in their sensitivity to variation in latencies of specific components under investigation. As our study attests, correcting for latency variability can help recover diminished ERP amplitudes, resulting in a more accurate interpretation of the data. We would therefore strongly encourage future ERP studies of older adults (and other participant populations) to assess and control for single trial variance—especially when making cross-sectional comparisons.

## Conclusion

Both behavioral and electrophysiological evidence now supports the view that recollection in older adults operates as a thresholded and variable process—a pattern similar to that observed in younger adults. Not only does our data provide a novel account of parietal retrieval success effects, it also highlights the potential problems with interpreting grand average ERP data in older adults. Indeed, our novel application of RIDE (a correction algorithm for single trial variance) allowed us to detect significant differences between conditions that were not detected before latency correction, and we would, therefore, encourage others to seriously consider the implications of latency variability when interpreting changes in ERP amplitude either between conditions or between groups. In the present case, a relatively subtle change in the pattern of effects reported across conditions has a significant impact on the theoretical interpretation of the findings. Analysis of uncorrected data failed to provide evidence of retrieval-related ERP effects, whereas significant effects were observed following RIDE, revealing a pattern between conditions that supports some-or-none accounts of recollection. Whilst the behavioral patterns of recollection are consistent with a some-or-none process in both younger and older adults, there was no reason to assume that similar underlying mechanisms supporting recollection would operate the same way. The present data clarify the functional significance of the parietal retrieval success effect in older adults, showing that is sensitive to the accuracy of recollected information. Given these findings, an important next step will be to understand how retrieval accuracy can be enhanced to improve memory quality in the elderly.

## Data Availability

The datasets generated for this study are available on request to the corresponding author.

## Ethics Statement

This study was carried out in accordance with the recommendations of the General University Ethics Panel. The protocol was approved by the General University Ethics Panel.

## Author Contributions

JM and DD conceptualized and designed the study. JM and GO performed the statistical analysis. JM wrote the first draft of the manuscript. All authors contributed to manuscript revision, read and approved the submitted version.

## Conflict of Interest Statement

The authors declare that the research was conducted in the absence of any commercial or financial relationships that could be construed as a potential conflict of interest.

## Footnotes

^1^The divisions between high and low accuracy trials were completely arbitrary—although the decision to bin the data this way provided us with an equal number of trials to form ERPs for each response category, whilst also reflecting the distribution of behavioral responses.

^2^For clarity, the article will make no specific reference to the R component—although more detail can be found in Ouyang et al. (2015a).

^3^https://bit.ly/30rNfly

^4^Although participants are given the opportunity to change their decision, our results suggest that a secondary decision is made very rarely (on average secondary decisions account for 3% of overall trials).
